# APELA Expression in Glioma, and Its Association with Patient Survival and Tumor Grade

**DOI:** 10.3390/ph12010045

**Published:** 2019-03-26

**Authors:** Debolina Ganguly, Chun Cai, Michelle M. Sims, Chuan He Yang, Matthew Thomas, Jinjun Cheng, Ali G. Saad, Lawrence M. Pfeffer

**Affiliations:** 1 Department of Pathology, University of Tennessee Health Science Center, Memphis, TN 38163, USA; dganguly@uthsc.edu (D.G.); ccai1@uthsc.edu (C.C.); msims7@uthsc.edu (M.M.S.); cyang@uthsc.edu (C.H.Y.); mthom101@uthsc.edu (M.T.); jinjun.cheng@nih.gov (J.C.); Ali.Saad@lebonheur.org (A.G.S.); 2 Department of Pathology and the Center for Cancer Research, University of Tennessee Health Science Center, Memphis, TN 38163, USA

**Keywords:** APELA, RNA-ISH, glioma, glioblastoma, cancer stem cells

## Abstract

Glioblastoma (GBM) is the most common and deadliest primary adult brain tumor. Invasion, resistance to therapy, and tumor recurrence in GBM can be attributed in part to brain tumor-initiating cells (BTICs). BTICs isolated from various patient-derived xenografts showed high expression of the poorly characterized Apelin early ligand A (APELA) gene. Although originally considered to be a non-coding gene, the APELA gene encodes a protein that binds to the Apelin receptor and promotes the growth of human embryonic stem cells and the formation of the embryonic vasculature. We found that both APELA mRNA and protein are expressed at high levels in a subset of brain tumor patients, and that APELA is also expressed in putative stem cell niche in GBM tumor tissue. Analysis of APELA and the Apelin receptor gene expression in brain tumor datasets showed that high APELA expression was associated with poor patient survival in both glioma and glioblastoma, and APELA expression correlated with glioma grade. In contrast, gene expression of the Apelin receptor or Apelin was not found to be associated with patient survival, or glioma grade. Consequently, APELA may play an important role in glioblastoma tumorigenesis and may be a future therapeutic target.

## 1. Introduction

Glioblastoma (GBM) is the most lethal intracranial neoplasm in adults and has a median survival of only 14–18 months [1]. Surgical resection combined with adjuvant chemotherapy and radiation therapy is the primary treatment of GBM, but has only provided slight improvement in the disease course and outcome for decades [2]. The cellular heterogeneity of GBM has been attributed to a subpopulation of brain tumor-initiating cells (BTICs), which express stem cell markers, can self-renew and undergo differentiation into multiple cell types [3,4,5]. BTICs have high tumor-initiating capacity and therapeutic resistance that can drive tumorigenesis and tumor recurrence after therapy [6]. We previously demonstrated that BTICs derived from patient-derived xenolines (PDXs) have markedly enhanced tumor-initiating activity when compared with BTICs induced to differentiate and form tumors that display identical histological features to the original PDX [7].

Apelin early ligand A (APELA), also called Elabela and Toddler, is an evolutionarily conserved peptide hormone that is essential for cardiovascular development in zebrafish through its binding to the G-protein coupled Apelin receptor (APLNR) [8]. APELA functions both as an endogenous ligand necessary for the growth of human embryonic stem cells [9] and in the formation of the embryonic vasculature [10]. Both Apelin and APELA bind to the Apelin receptor to transduce their distinct biological functions [9]. While the Apelin/APLNR pathway is associated with fluid homeostasis, the neuroendocrine response to stress, and cardiovascular function [11], the biological function of the APELA/APLNR pathway is poorly understood. In the present study, we found that Apelin early ligand A (APELA) gene expression was markedly upregulated in BTICs isolated from a number of PDXs when compared with BTICs induced to differentiate.

BTICs with high expression of the stem cell markers, Nestin and CD133, are localized near capillaries in brain tumors, and this vascular niche is apparently critical for stem cell renewal [12]. Since APELA [10] and the Apelin receptor [13] have been shown to play important roles in vascular development and stem cell proliferation, we examined whether APELA and APLNR were expressed in a stem cell niche in GBM patient tumor tissue by testing both RNA in situ hybridization (ISH) and immunohistochemistry (IHC), and found that APELA/APLNR-expressing cells were localized in a putative stem cell niche. Analysis of brain tumor expression datasets showed that high APELA expression was associated with poor patient survival. Our data suggest that APELA may play an important role in glioblastoma initiation and/or development, and could be a therapeutic target.

## 2. Results

### 2.1. Identification of APELA as a Gene Overexpressed in GBM and BTICs

**Figure 1:**
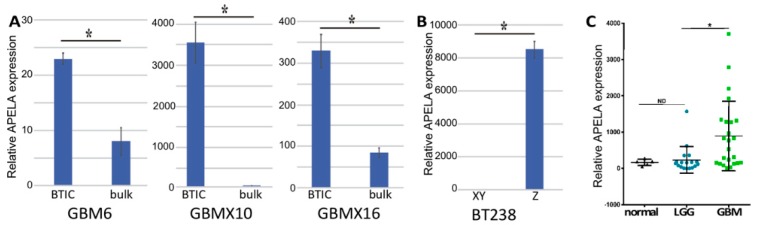
APELA gene expression in brain tumor-initiating cells (BTICs), and in normal brain and glioma samples. (**A**) APELA expression was determined by qPCR in RNA extracts from glioblastoma (GBM) patient-derived xenolines (PDXs) grown in bulk cultures or as BTICs, and (**B**) BTICs isolated from different regions of the BT238 tumor sample (n = 4). (**C**) RNA was extracted from FFPE blocks of normal brain tissue (5 samples), (LGG) low-grade glioma (18 samples) and (GBM) glioblastoma (24 samples), and APELA expression was measured by qPCR and normalized to ACTIN expression. * *p* < 0.05.

APELA was originally identified as a signaling protein in zebrafish embryos by database mining for non-annotated translated open reading frames [14]. Subsequently, APELA was found to promote both the growth of human embryonic stem cells and the formation of the embryonic vasculature [9,10]. To characterize the potential importance of APELA in stem-like cells in GBM, we examined APELA expression by qPCR in BTICs isolated from different GBM PDXs (GBM6, GBMX10, and GBMX16) and BTICs induced to differentiate. High APELA expression was found in BTICs isolated from various GBM PDXs as compared to BTICs induced to differentiate in the presence of serum (Figure 1A). As previously shown [15], serum-induced differentiation of BTICs increased the expression of the astrocyte marker glial fibrillary acidic protein (GFAP) and decreased the expression of neural stem cells markers, including Nestin and Sox2. In addition, significantly higher APELA expression was found in BTICs isolated from the central region of a GBM tumor (BT238Z) when compared with BTICs isolated from the tumor periphery (BT238XY) (Figure 1B).

Because APELA was overexpressed in BTICs, APELA expression was then determined by qPCR in RNA isolated from formalin-fixed paraffin-embedded (FFPE) biopsy specimens obtained from the University of Tennessee Health Science Center (UTHSC) tissue services core from primary low-grade glioma (LGG) (World Health Organization classification I and II), GBM (World Health Organization classification IV), and histologically benign brain samples. Although APELA was expressed at very low levels in normal brain samples, statistically significant higher APELA expression was found in GBM specimens (Figure 1C), which appeared to reflect a discrete subset of GBM patients with markedly higher APELA expression. 

### 2.2. APELA and Its Receptor APLNR Are Co-Expressed with the Stem Cell Marker Nestin in GBM Tumor Tissue

**Figure 2:**
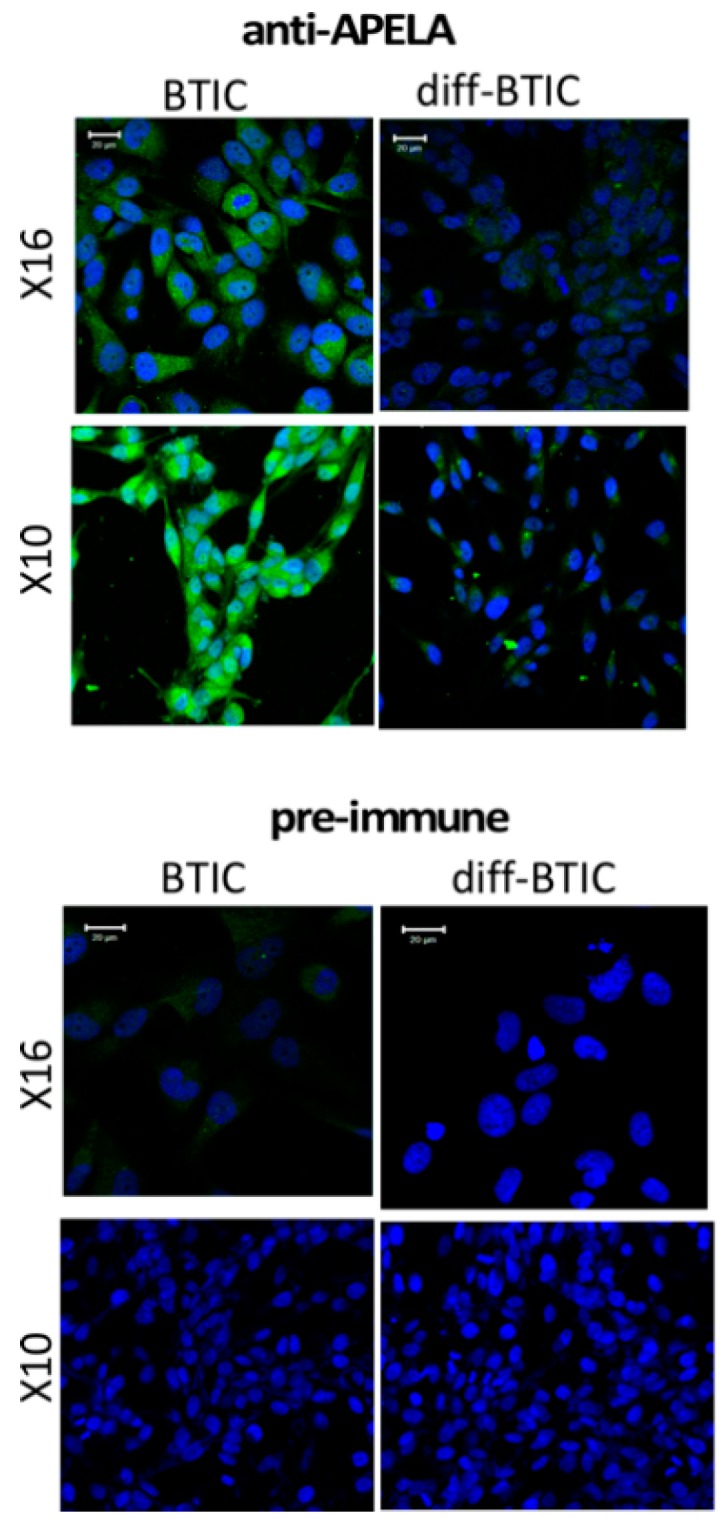
Immunohistochemistry (IHC) for APELA protein expression in BTICs and differentiated BTICs. IHC was performed on bulk differentiated and X10 and X16 BTICs. Staining was performed with APELA antisera or pre-immune sera and processed for IHC. Nuclei were counterstained with 4’,6-Diamidine-2’-phenylindole dihydrochloride (DAPI). Note, the markedly more intense cytoplasmic staining for APELA in BTICs than in bulk GBM cells, and no detectable staining obtained with pre-immune sera. Representative images are shown. Staining with pre-immune sera showed no IHC staining.

To characterize the expression of the APELA protein in GBM, antisera directed to the 34-amino acid APELA peptide was developed. GBMX10 and GBMX16 BTICs, and BTICs induced to differentiate in the presence of serum were grown on chamber slides and processed for IHC. As seen in Figure 2, robust APELA staining was observed in the cytoplasm of GBMX10 and GBMX16 BTICs as compared to BTICs that were induced to differentiate. Similar results were obtained with GBM6 BTICs (data not shown). As further evidence of the anti-APELA antibody’s specificity, no cellular staining was detected with pre-immune sera with either BTICs or differentiated BTICs. 

**Figure 3:**
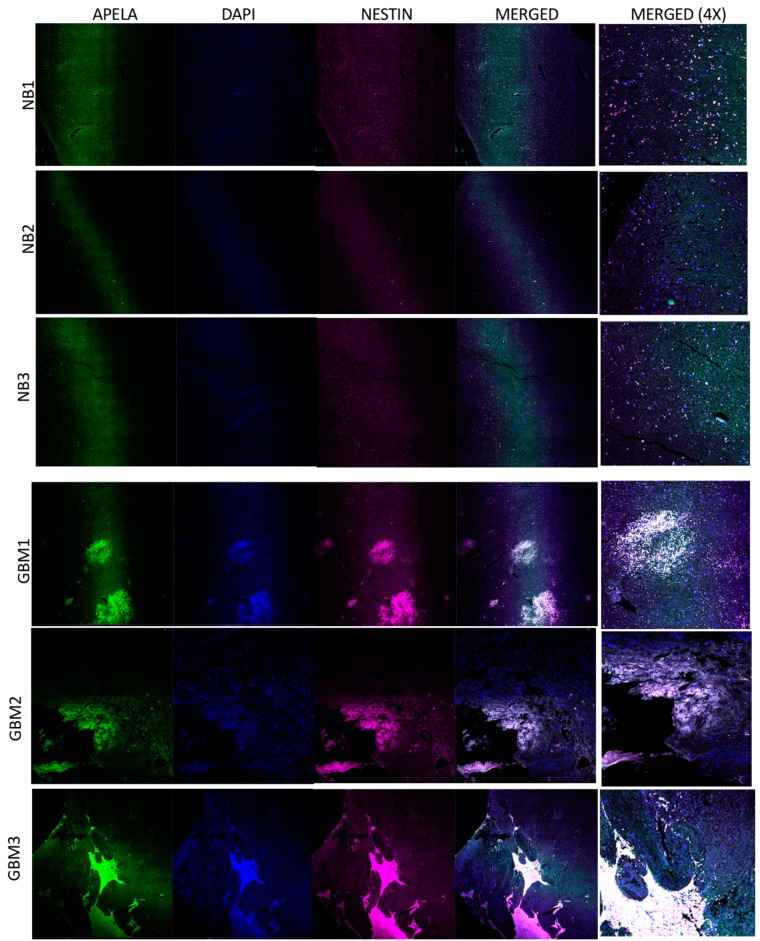
IHC for APELA protein expression in normal brain and glioma samples. IHC was performed on FFPE blocks of normal brain (NB) and GBM tumor tissue using anti-APELA and anti-Nestin Ab and processed for IHC. Representative images are shown.

To determine APELA protein expression in GBM patient specimens, we performed IHC on sections cut from FFPE tissue blocks of GBM patients and normal brain tissue. Since Nestin is a stem cell marker expressed in BTICs [16,17], we also determined whether APELA-expressing cells could be identified in a putative stem cell niche of Nestin-expressing cells by IHC using an anti-Nestin and anti-APELA specific antiserum. As shown in Figure 3 (lower three panels), Nestin staining was observed in a cluster of cells in three different GBM tumor samples that also showed APELA staining. In contrast, Nestin and APELA were rarely co-expressed in normal brain tissue by IHC.

**Figure 4:**
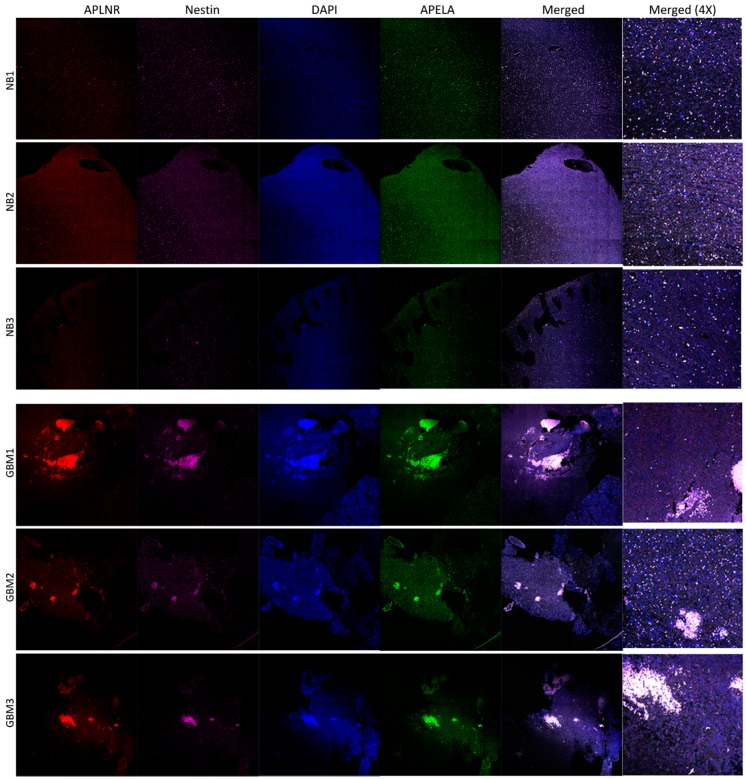
APELA is co-expressed with the stem cell marker Nestin at the mRNA level in GBM tumor tissue. Slides were prepared from normal brain (NB) and GBM tumor tissue, subjected to RNA-ISH using the RNAscope technology and images analyzed by confocal microscopy. RNA-ISH was performed with individual gene probes for APELA (green), and Nestin (magenta). Nuclei were DAPI counterstained (blue). Colocalization of APELA and Nestin expression in a cluster of individual cells is evident in the merged image (20× tile-scan magnification) and the 4× magnification of the merged image.

To determine APELA gene expression at the single cell level in human tissue, we performed RNA in situ hybridization (RNA-ISH) on sections cut from FFPE tissue blocks from GBM patient specimens and normal brain tissue. As shown in Figure 4, cells that co-expressed Nestin and APELA mRNA were found in discrete regions throughout tumor tissue from three different GBM patients. Moreover, cells that also expressed mRNA for the APELA receptor, APLNR, were also detectable by RNA-ISH in these discrete “nests” of tumor cells. Thus, similar patterns of Nestin and APELA co-expression were found at the mRNA level (RNA-ISH) and protein level (IHC) in GBM patient tumor specimens.

### 2.3. High APELA Expression is Associated with Poor Patient Survival in Glioma

**Figure 5:**
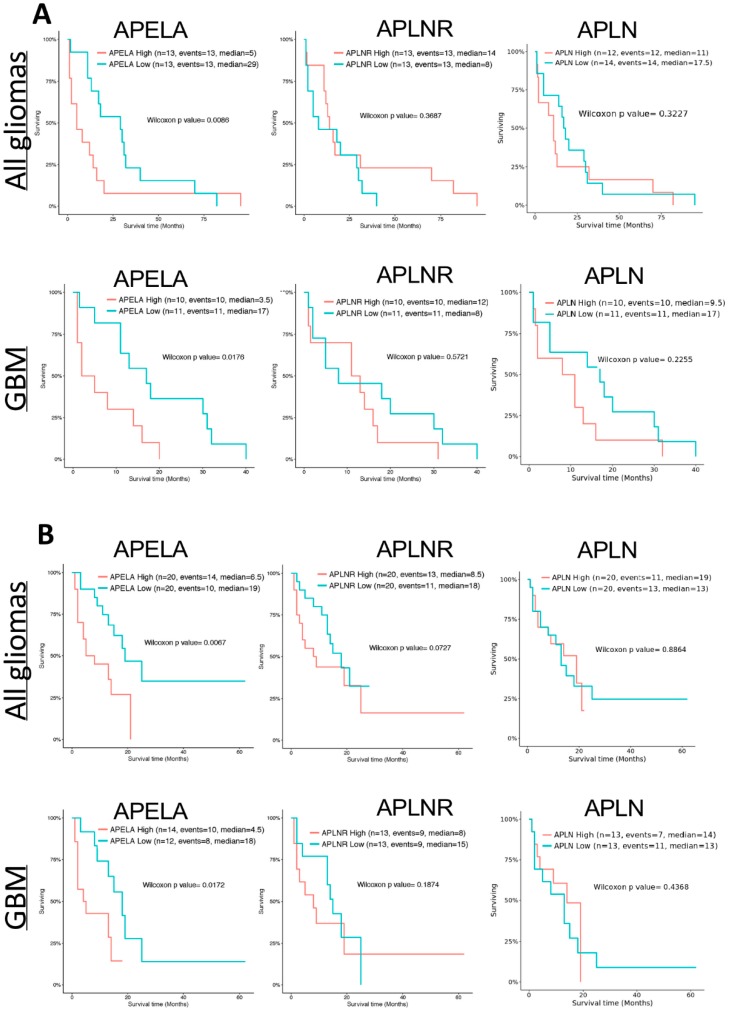
APELA expression is directly associated with glioma and GBM patient survival. (**A**,**B**) Kaplan–Meier curves of the association of APELA, APLNR and APLN expression with patient survival in the (**A**) Donson (GSE33331) [19] and (**B**) Vital (GSE43289) [20] microarray dataset analyzed using the GlioVis data portal [18].

**Figure 6:**
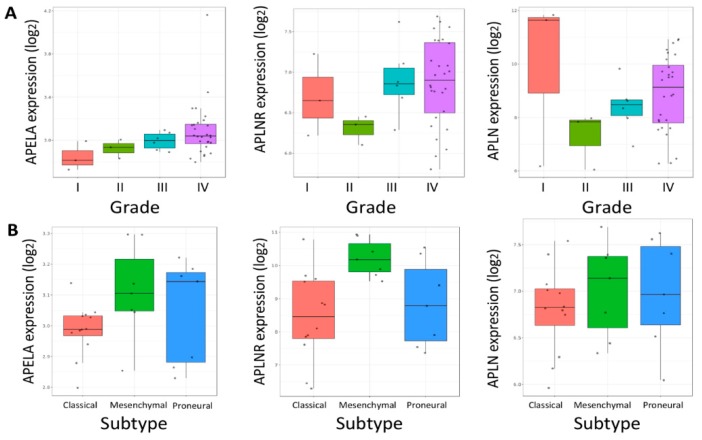
APELA expression as a function of glioma tumor grade and GBM molecular subtype. APELA, APLNR and APLN expression in the GSE43289 dataset plotted as a function of (**A**) histological grade in glioma or (**B**) molecular GBM subtype [20].

APELA was originally considered to be a noncoding transcript [14], and thus APELA expression data are absent from many of the gene expression databases of brain cancer tumors, such as The Cancer Genome Atlas (TCGA) and REMBRANDT (Repository for Molecular BRAin Neoplasia DaTa). The GlioVis data portal of brain tumor expression datasets [18] was used to examine the relationship between APELA and APLNR gene expression and patient survival. As shown in Figure 5, using two independent microarray datasets for glioma (GSE33331 and GSE43289) [19,20], high APELA expression was associated with poor patient survival (*p* value < 0.05), both when all grades of glioma were analyzed together and when GBM patients were analyzed independently. In contrast, neither the APELA receptor (APLNR) nor the other ligand that binds to this receptor Apelin (APLN) was associated with patient outcome in glioma or GBM (Figure 5). Moreover, when APELA expression from the GSE43289 dataset was analyzed based on the grade of the tumor tissue (Figure 6), although the number of samples analyzed in each group varied, the APELA expression was found to increase with tumor sample grade, with the highest APELA found in GBM (Grade IV). In contrast, expression of the Apelin receptor (APLNR) or Apelin (APLN) was not associated with any tumor grade (Figure 6A). Furthermore, APELA expression was expressed in all the molecular subtypes of GBM, with somewhat higher APELA expression observed in both the proneural and mesenchymal subtypes (Figure 6B) Taken together, the close relationship of APELA expression with brain tumor patient survival and tumor grade at diagnosis suggests that APELA plays an important role in glioma pathogenesis.

## 3. Discussion

In this study, we identified that APELA mRNA was overexpressed in BTICs that are isolated from several PDXs compared to differentiated BTICs. It is important to note that these PDXs were all defined as being of the classical GBM subtype by molecular characterization [21]. However, recent studies using single cell RNA sequencing have found that cells of the different GBM molecular subtypes are present within a single GBM tumor [22]. Our findings in the BT238 GBM PDX are consistent with the molecular heterogeneity of GBM tumors, since we found a significant variation in APELA expression in BTICs derived from different regions of the same GBM tumor (BT238XY versus BT238Z).

In addition, we found that APELA gene expression was significantly overexpressed in RNA isolated from FFPE blocks of GBM patient tissue compared to low-grade glioma tissue and normal brain tissue. To further characterize the role of APELA in GBM, we developed a highly specific rabbit antisera directed against the 34-amino acid secreted form of APELA. Similar to our results at the RNA level, we found that the APELA protein was expressed at higher levels in BTICs through immunohistochemical staining compared to differentiated BTICs. In contrast, pre-immune sera showed no immunoreactivity against BTICs, confirming that our anti-APELA was highly specific. Furthermore, by using RNA-ISH probes, we showed that APELA mRNA was expressed both in GBM tumor tissue and most interestingly in cells that co-express the stem cell marker Nestin. However, APELA gene expression was negligible in normal brain tissue. Furthermore, using IHC, we found a significant overlap in cells that express both Nestin and APELA in GBM tumor tissue. Taken together, our results suggest that APELA is expressed in a stem cell niche within GBM tumor tissue. In previous studies, BTICs with high expression of the stem cell markers Nestin and CD133 were localized near capillaries in brain tumors, suggesting that the tumor microenvironment may influence BTIC function [12]. Moreover, consistent with our findings, increased APELA immunoreactivity was observed in high-grade glioma when compared to that of low-grade glioma using a commercially available anti-APELA antibody [23]. Interestingly, APELA levels have also been quantified in sera from patients with pre-eclampsia [24]. In future studies, it would be of interest to quantify APELA levels in the sera from brain tumor patients and determine whether APELA may serve as a biomarker for patient outcome as well as response to therapy.

APELA is an evolutionary conserved secreted peptide hormone that binds to the Apelin receptor (APLNR) to mediate endoderm differentiation during zebrafish embryogenesis [8,14]. In humans, APELA is first detected in pre-implantation blastocysts and controls the self-renewal of embryonic stem cells [9]. In the human adult, APELA expression is mainly restricted to endocrine organs including the kidney and placenta [9], and has been shown to play an important role in pre-eclampsia and cardiovascular malformations in mice [24]. Besides binding APELA, the Apelin receptor also binds the peptide hormone Apelin to transduce biological functions [9]. The Apelin/APLNR system mediates heart contractility and blood pressure regulation [25,26]. Recent studies suggest that the APLNR/Apelin system may play an important role in tumorigenesis. Pharmacological targeting of APLNR impaired the growth of glioblastoma cells in vitro and in GBM xenografts in vivo [27]. Consistent with these findings, we found that cells that expressed APELA mRNA in GBM tumor tissue also expressed APLNR. Future studies should focus on addressing whether Apelin and APELA play distinct roles in promoting GBM tumorigenesis. 

To explore the potential role of APELA in glioma, we analyzed microarray data from two different glioma gene expression studies for the relationship between APELA expression and patient survival. Interestingly, in glioma patients there was a significant difference in patient survival between high APELA expressing patients and low APELA expressing patients, with high APELA being associated with lower patient survival. Moreover, when GBM patients were analyzed for APELA expression there was also a close association with high APELA expression and poor patient survival. In addition, APELA expression tended to increase in accord with the histological grade of the glioma. In contrast, we found that expression of the receptor for APELA, APLNR, did not show any correlation with glioma patient survival. In addition, our qPCR data on APELA expression on FFPE tissue correlated well with the microarray database analysis, since we found higher APELA expression in GBM than in low-grade glioma. These results are consistent with APELA promoting glioma tumorigenesis. Interestingly, preliminary database analysis suggests that APELA maybe overexpressed in other human malignancies. For example, APELA has been found to be overexpressed ovarian cancer, and APELA promoted cell growth and cell cycle progression in ovarian cancer cell lines [28]. Thus, future studies should focus on the role of APELA in these tumor types and whether APELA may be an important target for therapeutic intervention.

## 4. Materials and Methods

**Cell Culture:** GBM6, GBMX10 and GBMX16 (obtained from Dr. Y. Gillespie, Department of Neurosurgery, University of Alabama at Birmingham), and BT238XY and BT238Z (obtained from Dr. Samuel Weiss, Cumming School of Medicine, University of Calgary, Alberta) patient-derived xenolines (PDXs) were maintained as xenografts in immunocompromised mice [29]. These PDXs were defined as being the classical subtype of GBM using molecular characterization [21]. BTICs were isolated, maintained in flasks pre-coated with poly-D-lysine and laminin, and grown in a NeuroBasal-A medium (Invitrogen, Carlsbad, CA, USA) containing 2% B27 supplement, 2 mM l-glutamine, 100 units/mL penicillin, 100 g/mL streptomycin, epidermal growth factor (20 ng/mL), and basic fibroblast growth factor (40 ng/mL), as described in previous studies [15,29].

**Gene Expression Analysis:** Gene expression was determined in RNA isolated from de-identified formalin-fixed paraffin-embedded (FFPE) patient biopsy specimens (UTHSC Tissue Services Core) as previously described [30]. In brief, total RNA was extracted using the QIAshredder and RNeasy mini kits (Qiagen Inc., Frederick, MD, USA). Quantitative real time PCR (qPCR) was performed using gene-specific primers for APELA (forward 5′-CGAGTGCCCTTCCCATGA-3′ and reverse 5′-TTGCTTTCACCCTTCTTCTGGTA-3′) and BETA-ACTIN (forward 5′-GGACTTCGAGCAAGAGATGG-3′ and reverse 5′-AGCACTGTGTTGGCGTACAG-3′) with an iScript one-step RT-PCR kit containing SYBR Green (Bio-Rad, Hercules, CA, USA). The reaction parameters were as follows: cDNA synthesis at 50 °C for 20 min, transcriptase inactivation at 95 °C for 5 min, and PCR cycling at 95 °C for 10 s and 60 °C for 30 s for 40 cycles. APELA gene expression was normalized relative to ACTIN expression.

**Anti-APELA Antibodies:** Antisera was produced in rabbits immunized against the 34-amino acid secreted form of APELA according to the manufacturer’s protocol (ThermoFisher Scientific, Waltham, MA, USA). Antisera collected between 28 to 72 days post-inoculation was found to display immunoreactivity against the APELA peptide at >1:1000 dilution as assessed by ELISA, while pre-immune sera was not immunoreactive.

**Immunohistochemistry (IHC):** For IHC on BTICs and BTICs induced to differentiate, cells were grown in 8-well chamber slides (Millipore, Burlington, MA, USA) precoated with poly-d-lysine and laminin to ~50% confluency, fixed with 4% paraformaldehyde, and permeabilized with Triton X-100. For IHC on FFPE tissue, blocks were cut into 5-μm sections, affixed onto glass slides, deparaffinized, and treated for target retrieval according to the RNA-ISH protocol. After blocking with 5% goat serum, slides for IHC were incubated with antibodies against Nestin (1:400 dilution, Abcam, Cambridge, MA, USA) and APELA (1:100 dilution), and subsequently stained with Alexa Fluor 488 or 594 (Molecular Probes, Carlsbad, CA, USA). Images were captured on a laser-scanning confocal microscope at 20x tile-scan magnification (Zeiss model LSM700, San Diego, CA, USA).

**RNA-ISH:** For RNA-ISH on FFPE tissue, 5-μm sections on glass slides were baked for 1 h at 60 °C, deparaffinized, and treated for target retrieval according to the manufacturer’s protocol. The FFPE sections were then incubated with RNAscope ISH probes (Advanced Cell Diagnostics, Newark, DE, USA) and hybridized sequentially to visualize target RNA signals, according to the RNAscope Fluorescent Multiplex Kit user manual. Images were captured on a laser-scanning confocal microscope.

**Tumor Xenografts in Mice:** All animal experiments were performed in accordance with protocol #17-098.0-A and were approved by the Institutional Animal Care and Use Committee of the University of Tennessee Health Science Center. BTICs were dissociated with HyQTase, resuspended in PBS, and xenografts were established in 4–6-week-old male NOD.Cg-Prkdc^scid^ Il2rgtm1Wjl/SzJ (NSG) mice (Jackson Laboratory, Bar Harbor, ME, USA) by injecting X10 BTICs (10^6^ cells/100 µL PBS) into the flanks. Tumor burden was assessed weekly by palpation with slide caliper measurement. Once tumors reached ~1000 mm^3^, mice were euthanized, and tumors were either minced and serially passaged or frozen at −80 °C in cryomolds for IHC and RNA-ISH.

**Analysis of Brain Tumor Expression Datasets:** Microarray data from two datasets that included APELA and APLNR gene expression using U133Plus2.0 chips and related patient survival [19,20] were analyzed using the GloVis platform (http://gliovis.bioinfo.cnio.es/) [18]. Data was plotted based on the histopathology assessment of the brain tumor tissue.

**Data Analysis:** At least three independent experiments were performed in triplicate, and data are presented as mean ± SEM. Analysis of variance or Student’s t-tests were performed and *p* ≤ 0.05 was considered to be statistically significant.

## 5. Conclusions

In conclusion, we demonstrated that the poorly characterized APELA gene is dysregulated at the mRNA and protein level in brain cancer, and that APELA expression is correlated with pathological tumor grade and poor survival. We hypothesize that APELA plays an important but poorly understood role in glioblastoma initiation and/or development.

## References

[1] Surawicz T.S., Davis F., Freels S., Laws E.R., Menck H.R. (1998). Brain tumor survival: Results from the national cancer data base. J. Neuro-Oncol..

[2] Stupp R., Mason W.P., van den Bent M.J., Weller M., Fisher B., Taphoorn M.J., Belanger K., Brandes A.A., Marosi C., Bogdahn U. (2005). Radiotherapy plus concomitant and adjuvant temozolomide for glioblastoma. N. Engl. J. Med..

[3] Clarke M.F., Dick J.E., Dirks P.B., Eaves C.J., Jamieson C.H., Jones D.L., Visvader J., Weissman I.L., Wahl G.M. (2006). Cancer stem cells–perspectives on current status and future directions: Aacr workshop on cancer stem cells. Cancer Res..

[4] Mani S.A., Guo W., Liao M.J., Eaton E.N., Ayyanan A., Zhou A.Y., Brooks M., Reinhard F., Zhang C.C., Shipitsin M. (2008). The epithelial-mesenchymal transition generates cells with properties of stem cells. Cell.

[5] Marotta L.L., Polyak K. (2009). Cancer stem cells: A model in the making. Curr. Opin. Genet. Dev..

[6] Huntly B.J., Gilliland D.G. (2005). Leukaemia stem cells and the evolution of cancer-stem-cell research. Nat. Rev. Cancer.

[7] Pollard S.M., Yoshikawa K., Clarke I.D., Danovi D., Stricker S., Russell R., Bayani J., Head R., Lee M., Bernstein M. (2009). Glioma stem cell lines expanded in adherent culture have tumor-specific phenotypes and are suitable for chemical and genetic screens. Cell Stem Cell.

[8] Chng S.C., Ho L., Tian J., Reversade B. (2013). Elabela: A hormone essential for heart development signals via the apelin receptor. Dev. Cell.

[9] Ho L., Tan S.Y., Wee S., Wu Y., Tan S.J., Ramakrishna N.B., Chng S.C., Nama S., Szczerbinska I., Chan Y.S. (2015). Elabela is an endogenous growth factor that sustains hesc self-renewal via the pi3k/akt pathway. Cell Stem Cell.

[10] Helker C.S., Schuermann A., Pollmann C., Chng S.C., Kiefer F., Reversade B., Herzog W. (2015). The hormonal peptide elabela guides angioblasts to the midline during vasculogenesis. Elife.

[11] O’Carroll A.M., Lolait S.J., Harris L.E., Pope G.R. (2013). The apelin receptor apj: Journey from an orphan to a multifaceted regulator of homeostasis. J. Endocrinol..

[12] Calabrese C., Poppleton H., Kocak M., Hogg T.L., Fuller C., Hamner B., Oh E.Y., Gaber M.W., Finklestein D., Allen M. (2007). A perivascular niche for brain tumor stem cells. Cancer Cell.

[13] Liu Q., Hu T., He L., Huang X., Tian X., Zhang H., He L., Pu W., Zhang L., Sun H. (2015). Genetic targeting of sprouting angiogenesis using apln-creer. Nat. Commun..

[14] Pauli A., Norris M.L., Valen E., Chew G.L., Gagnon J.A., Zimmerman S., Mitchell A., Ma J., Dubrulle J., Reyon D. (2014). Toddler: An embryonic signal that promotes cell movement via apelin receptors. Science.

[15] Ganguly D., Fan M., Yang C.H., Zbytek B., Finkelstein D., Roussel M.F., Pfeffer L.M. (2018). The critical role that Stat3 plays in glioma-initiating cells: Stat3 addiction in glioma. Oncotarget.

[16] Garner J.M., Fan M., Yang C.H., Du Z., Sims M., Davidoff A.M., Pfeffer L.M. (2013). Constitutive activation of signal transducer and activator of transcription 3 (Stat3) and nuclear factor kappa beta signaling in glioblastoma cancer stem cells regulates the notch pathway. J. Biol. Chem..

[17] Ganguly D., Sims M., Cai C., Fan M., Pfeffer L.M. (2018). Chromatin remodeling factor brg1 regulates stemness and chemosensitivity of glioma initiating cells. Stem Cells.

[18] Bowman R.L., Wang Q., Carro A., Verhaak R.G., Squatrito M. (2017). Gliovis data portal for visualization and analysis of brain tumor expression datasets. Neuro Oncol..

[19] Donson A.M., Birks D.K., Schittone S.A., Kleinschmidt-DeMasters B.K., Sun D.Y., Hemenway M.F., Handler M.H., Waziri A.E., Wang M., Foreman N.K. (2012). Increased immune gene expression and immune cell infiltration in high-grade astrocytoma distinguish long-term from short-term survivors. J. Immunol..

[20] Vital A.L., Tabernero M.D., Castrillo A., Rebelo O., Tao H., Gomes F., Nieto A.B., Resende Oliveira C., Lopes M.C., Orfao A. (2010). Gene expression profiles of human glioblastomas are associated with both tumor cytogenetics and histopathology. Neuro Oncol..

[21] Verhaak R.G., Hoadley K.A., Purdom E., Wang V., Qi Y., Wilkerson M.D., Miller C.R., Ding L., Golub T., Mesirov J.P. (2010). Integrated genomic analysis identifies clinically relevant subtypes of glioblastoma characterized by abnormalities in pdgfra, idh1, egfr, and nf1. Cancer Cell.

[22] Patel A.P., Tirosh I., Trombetta J.J., Shalek A.K., Gillespie S.M., Wakimoto H., Cahill D.P., Nahed B.V., Curry W.T., Martuza R.L. (2014). Single-cell rna-seq highlights intratumoral heterogeneity in primary glioblastoma. Science.

[23] Artas G., Ozturk S., Kuloglu T., Dagli A.F., Gonen M., Artas H., Aydin S., Erol F.S. (2018). A novel candidate molecule in the pathological grading of gliomas: Elabela. Turk. Neurosurg..

[24] Ho L., van Dijk M., Chye S.T.J., Messerschmidt D.M., Chng S.C., Ong S., Yi L.K., Boussata S., Goh G.H., Afink G.B. (2017). Elabela deficiency promotes preeclampsia and cardiovascular malformations in mice. Science.

[25] Dai T., Ramirez-Correa G., Gao W.D. (2006). Apelin increases contractility in failing cardiac muscle. Eur. J. Pharmacol..

[26] Ashley E.A., Powers J., Chen M., Kundu R., Finsterbach T., Caffarelli A., Deng A., Eichhorn J., Mahajan R., Agrawal R. (2005). The endogenous peptide apelin potently improves cardiac contractility and reduces cardiac loading in vivo. Cardiovasc. Res..

[27] Harford-Wright E., Andre-Gregoire G., Jacobs K.A., Treps L., Le Gonidec S., Leclair H.M., Gonzalez-Diest S., Roux Q., Guillonneau F., Loussouarn D. (2017). Pharmacological targeting of apelin impairs glioblastoma growth. Brain J. Neurol..

[28] Yi Y., Tsai S.H., Cheng J.C., Wang E.Y., Anglesio M.S., Cochrane D.R., Fuller M., Gibb E.A., Wei W., Huntsman D.G. (2017). Apela promotes tumour growth and cell migration in ovarian cancer in a p53-dependent manner. Gynecol. Oncol..

[29] Garner J.M., Ellison D.W., Finkelstein D., Ganguly D., Du Z., Sims M., Yang C.H., Interiano R.B., Davidoff A.M., Pfeffer L.M. (2015). Molecular heterogeneity in a patient-derived glioblastoma xenoline is regulated by different cancer stem cell populations. PLoS ONE.

[30] Yang C.H., Yue J., Pfeffer S.R., Fan M., Paulus E., Hosni-Ahmed A., Sims M., Qayyum S., Davidoff A.M., Handorf C.R. (2014). Microrna-21 promotes glioblastoma tumorigenesis by down-regulating insulin-like growth factor-binding protein-3 (igfbp3). J. Biol. Chem..

